# Supplementary material for: Constructing functional models from biophysically-detailed neurons
Source: PLoS Comput Biol. 2022 Sep 8;18(9):e1010461. doi: 10.1371/journal.pcbi.1010461 (PMC9455888; doi:10.1371/journal.pcbi.1010461)
Supplement: S1 Appendix — Includes a table that reports the time constants used for each of the networks in Sec 4, as well as a figure that reports the distribution of time constants obtained when repeatedly running Hyperopt. (PDF) [file pcbi.1010461.s001.pdf]

S1 Appendix: Synaptic Time Constants

Table A reports the time constants (in ms) obtained using Hyperopt for each of the networks used in the Results, with τ_1 corresponding to the synaptic rise time and τ_2 corresponding to the fall time in a double exponential filter. Note that most networks have τ_1 near 30ms and τ_2 between 100ms and 200ms. We observed that our synaptic optimization tends to return filters with large rise time, as these filters smooth spiking activity into a more continuous signal that can be easily decoded to the target function. The proximity of most τ_1 to 30ms reflects an upper-bound we placed on τ_1 in our optimization code; lowering this upper bound produces synapses with more realistic time constants but degrades the performance of our networks somewhat. Similarly, most τ_2 are above 100ms because the oracle stream used a default filter with τ_{fall} =100ms; to match the degree of filtering that these synapses imposed on the state space and spike space targets, our optimized filters typically needed similarly long decay times.

Table A. Synaptic time constants obtained by Hyperopt for the networks described in Sec. 4.1-4.2.

	**LIF**	**Izhikevich**	**Wilson**	**Pyramidal**
Adaptation	$\tau_1 = 28.5,\ \tau_2 = 102$	$\tau_1 = 26.9,\ \tau_2 = 144$	$\tau_1 = 29.4,\ \tau_2 = 191$	$\tau_1 = 17.8,\ \tau_2 = 141$
Identity	$\tau_1 = 27.2,\ \tau_2 = 96.7$	$\tau_1 = 26.9,\ \tau_2 = 144$	$\tau_1 = 30.0,\ \tau_2 = 174$	$\tau_1 = 13.3,\ \tau_2 = 139$
Multiplication	$\tau_1 = 29.4,\ \tau_2 = 97.8$	$\tau_1 = 29.9,\ \tau_2 = 135$	$\tau_1 = 26.9,\ \tau_2 = 179$	$\tau_1 = 28.6,\ \tau_2 = 129$
Harmonic Oscillator	$\tau_1 = 29.2,\ \tau_2 = 118$	$\tau_1 = 29.9,\ \tau_2 = 172$	$\tau_1 = 27.8,\ \tau_2 = 184$	$\tau_1 = 26.3,\ \tau_2 = 136$
Difference memory	$\tau_1 = 28.3,\ \tau_2 = 67.2$	$\tau_1 = 28.5,\ \tau_2 = 106$	$\tau_1 = 29.8,\ \tau_2 = 148$	$\tau_1 = 20.0,\ \tau_2 = 147$

Fig A reports the distribution of time constants (in ms) obtained when repeatedly running Hyperopt. The data are generated using the network shown in Fig 2 of the main text: encoders are trained once using Eq. 8-9, after which spikes are collected by simulating the network with 10 different white noise signals. The Hyperopt optimization described in Sec. 3.3 is then performed on this data with 100 different random seeds. demonstrates that the random search used by osNEF to find synaptic parameters reliably finds time constants within a narrow range of values.

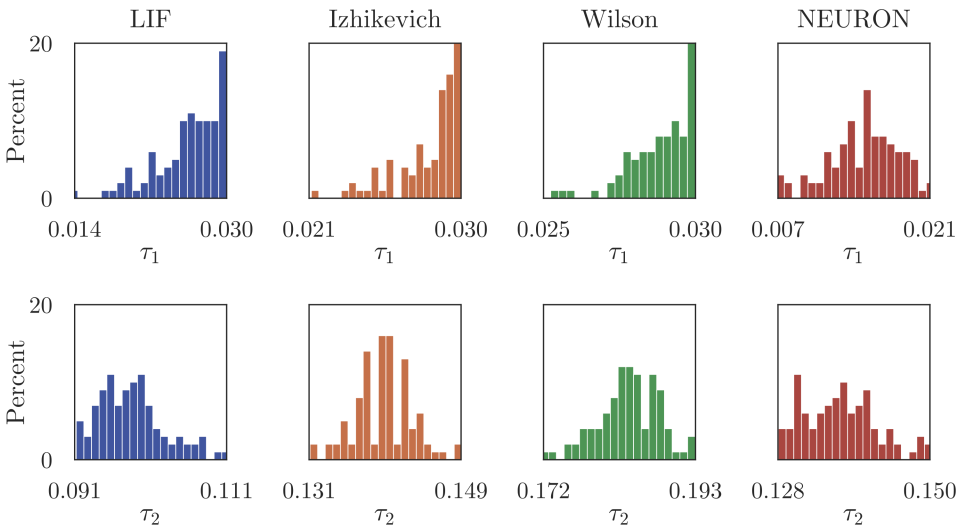

Fig A. Distribution of synaptic time constants obtained by repeated search using Hyperopt.
