# Supplementary material for: Constructing functional models from biophysically-detailed neurons
Source: PLoS Comput Biol. 2022 Sep 8;18(9):e1010461. doi: 10.1371/journal.pcbi.1010461 (PMC9455888; doi:10.1371/journal.pcbi.1010461)
Supplement: S2 Appendix — Includes an additional experiment that investigates how the performance of osNEF-trained networks degrades as external noise is introduced into the system. (PDF) [file pcbi.1010461.s002.pdf]

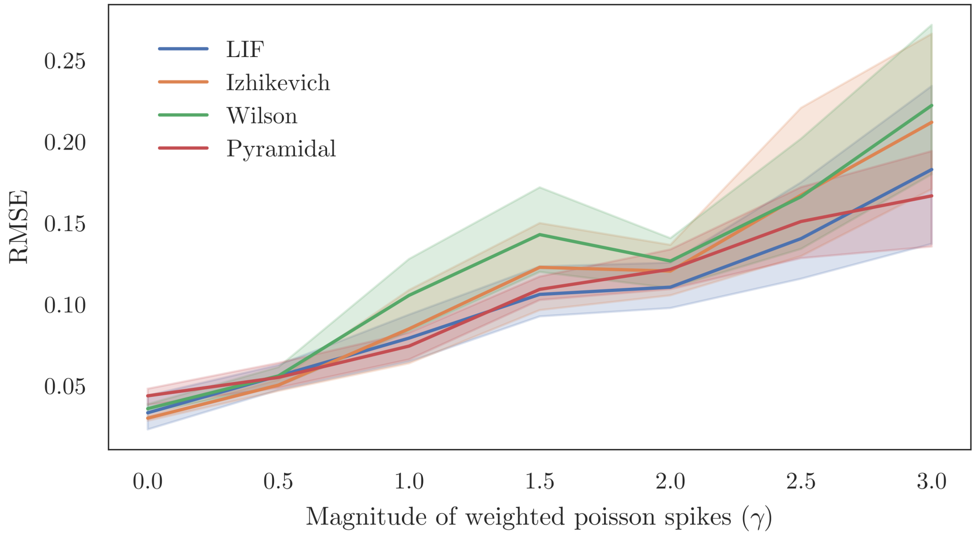

Fig A. Performance when computing the identity function degrades gracefully as external noise (in the form poisson spikes) is applied with increasing weight. Shaded regions are 95% confidence intervals over tests with unique input signals and weight matrices.

S2 Appendix: Performance versus Noise

How does the performance of osNEF-trained networks degrade as external noise is introduced into the system? To investigate this question, we took the network in Fig 5 of the main text and introduced a population of Poisson spiking neurons, "poisson", that are driven by a 10Hz band-limited white noise signal. We connected "poisson" to "pop_1" with a random weight matrix $w^{\text{poisson-pop}}$ that was obtained by copying $w^{\text{pre-pop}}$ and randomly shuffling the entries. By setting the maximum firing rates of "poisson" to mirror the maximum firing rates of "pre", and setting $w^{\text{poisson-pop}}$ to be a shuffled version of the osNEF-trained $w^{\text{pre-pop}}$, we ensured that the weighted spikes delivered to "pop_1" by "poisson" would drive our detailed neurons with (approximately) the same force as a standard state space input. We then scaled $w^{\text{poisson-pop}}$ by a factor γ between zero and three: $\gamma = 0$ causes noise having no effect, $\gamma = 1$ causes noise to have (approximately) the same impact as the input $\mathbf{x}(t)$, and $\gamma > 1$ should cause noise to overwhelm the input signal. For each neuron model and for each value of γ, we ran five tests with unique input signals, noise signals, and random shufflings of $w^{\text{poisson-pop}}$. Fig A plots the change in RMSE between the decoded estimate from "pop_2" and the state space target as a function of γ. As expected, RMSE grows as the impact external noise becomes greater; this increase appears to be approximately linear in γ, which is a good indication that our representational scheme is robust to external perturbation and degrades gracefully as the signal-to-noise ratio decreases.
