# Supplementary material for: Constructing functional models from biophysically-detailed neurons
Source: PLoS Comput Biol. 2022 Sep 8;18(9):e1010461. doi: 10.1371/journal.pcbi.1010461 (PMC9455888; doi:10.1371/journal.pcbi.1010461)
Supplement: Attachment — Submitted filename: Response to Reviewers.docx [file pcbi.1010461.s003.docx]

Dear Editor and Reviewers,

Thank you for the detailed feedback you provided on our submission entitled “Constructing Functional Models from Biophysically-Detailed Neurons” in PLOS Computational Biology. We found your comments quite helpful, and believe the revised paper includes clearer methodological explanation, more detailed analyses of simulations, and broader discussion of our methods relative to other contemporary approaches. In this letter, we detail the changes we have made to our submission. We hope that, with these significant revisions, our paper will continue to be considered for publication in PLOS Comp Bio.

Reviewer 1

Reviewer 1 requested that (a) several experiments/analyses be added to the paper, and (b) several modeling choices and explanations be clarified. We have incorporated the majority of these suggestions into either the main paper or appendices.

**It’s somewhat frustrating that there’s no name for the method to train biophysically detailed neuron models. I understand that this technique borrows from many sources, such as the NEF, but it’s hard to keep focus when it’s just referred to in third person and never by any proper name like “Time NEF” or “encoder-training boosted NEF”.**

We acknowledge that naming our method will make it easier to refer to, in the text and in the wider community. We have named the method “oracle supervised Neural Engineering Framework” (osNEF) to indicate that (a) the method is primarily an extension of core NEF principles, (b) the method includes an ‘oracle’, a parallel network that is externally driven to exhibit the target state-space dynamics, and which consequently generates the target spike-space activity patterns. The ‘oracle’ uses both state space and spike space ‘supervision’ to train encoders and decoders in the main networks. This naming, and the rationale behind it, are described in the introduction, and consistently used to identify our methods, especially when comparing them to other approaches.

**I think this paper would benefit substantially from a numerical demonstration of a non-linear dynamical system. There are techniques (such as the efficient coding based spiking networks) for which non-linear dynamics are difficult. Some justification/testing is required for the statement “the method remains the same for nonlinear system”. In particular, I have some doubts that the non-linear dynamics of something like the Lorenz or Rosler systems will be reproduced when the neural tuning curves are approximated by ReLus, but I could be wrong.**

The reviewer is correct that many training methods struggle when applied to nonlinear systems. Our claim is not that osNEF will successfully realize such systems, but that there are no mathematical assumptions in our theory or training techniques that require the target system to be linear. Supporting the larger claim would indeed require more extensive justification and testing. Previous work has shown that standard NEF techniques can realize the Lorenz attractor with populations of LIF neurons (e.g., [this tutorial](https://www.nengo.ai/nengo/examples/dynamics/lorenz-attractor.html)). We have also experimented with applying osNEF to train this system in the last few weeks; we have found that the osNEF filter and decoder optimization may be used to realize the Lorenz attractor in a population of 100 ReLU or LIF neurons, whose encoders and biases are set using standard NEF tools. However, we have thus far been unable to remove biases and use osNEF’s online encoder learning rule to reproduce these results, or to apply this training to the other, more detailed neuron models. This is likely an issue with numerical stability and precision in this 3D chaotic system, an area where osNEF struggles relative to standard NEF. With the deadline for revised resubmission approaching, we have listed this as a goal for future research, and changed the language in the paper to be more conservative regarding osNEF’s applicability to nonlinear systems.

**The choice of the ReLU. Why use a ReLu vs a type-I tuning curve? These are the tuning curves that would be expected for type-I neurons on a SNIC bifurcation. Also, the LIF neuron model here seems a weird choice, since there’s already a derivable tuning curve.**

The choice of target tuning curve in osNEF is arbitrary. We chose ReLUs for this paper, instead of a type-1 tuning curve, for two reasons. First, ReLUs are the simplest spiking neurons that still capture an essential neural nonlinearity: the transition from a region (in state space) where the neuron remains (mostly) inactive, to a region where activity increases monotonically (as state inputs change). The parameters inherent to ReLUs also correspond intuitively to the x- and y-intercepts of a tuning curve; this is important because standard NEF techniques choose neural parameters such that the distribution of target tuning curves have a variety of x- and y-intercepts, which ensures that a neural population robustly represents all regions of a state space. We felt that these two features of ReLUs facilitated a straightforward explanation of these underlying concepts for readers who are less familiar with neuron models and electrophysiological realism. Still, we have expanded our discussion of tuning curves and ReLUs in section 3.1 to justify this choice, notably by highlighting the relationship between the above nonlinearity, tuning curve distributions, and representational accuracy.

The second reason that we chose ReLUs instead of a type-1 neuron (such as LIF) is that both neuron models work as target tuning curves in the systems and firing rate regimes we investigate. The reviewer is correct that the activity of detailed neuron models will be poorly approximated by the ReLU neuron in many regimes, notably for high firing rates, where ReLU activities increase linearly without end while realistic neuron activities will plateau. However, we observed these differences to be negligible in our simulations for two reasons, which are both evident in Figure 3 (which plots neural activity as a function of input state for the ReLU and four trained neuron models). First, for the state spaces that we simulate, and for the maximum firing rates we target, the differences between a ReLU and a LIF tuning curve are minimal: in Figure 3, ReLU activities only exceed LIF activities for extreme inputs (**x**>0.8), and these differences fall within the measured confidence intervals of the ReLU and LIF tuning curves. (Recall that confidence intervals arise from noisy spike trains in dynamic simulations, and from converting spikes to real-valued firing rate via convolution with a smoothing filter). Second, osNEF training of encoders for a given neuron model is fundamentally constrained by the electrophysiology of that neuron model. Because osNEF training minimizes the differences between spiking activity of a trained neuron and a target neuron, trained neurons will often match the “physiologically plausible” features of a target tuning curve but fail to match any “physiologically implausible” features. This tendency can also be observed in Figure 3: for most inputs (**x**<0.8), our trained neurons qualitatively match the inactive and linear segments of the target ReLU curve, but for extreme inputs (**x**>0.8), our trained neurons begin to plateau, failing to match the target tuning curve but retaining their realistic response properties. Ultimately, any target tuning curve will have different response properties than the neuron model being trained; we felt that, by choosing a target tuning curve that was manifestly *implausible* in some respects, then showing that our trained neuron models still achieved the desired response properties, we demonstrated that osNEF does not require perfect selection of target tuning curves.

Finally, we ran several experiments using LIFs as the target tuning curves instead of ReLUs, and observed similar performance across both neuron models and network function. Additionally, we have revised Sections 3.1 (tuning curves) and 4.1 (representation, including Figure 3) to more thoroughly justify our choice of ReLUs, as per the above points.

**Because the synaptic time constants were part of the optimization, I would like to see a table that shows their values for all tasks considered. This partially shown in the representation task but we need a table for every distinct network trained/task, since the time constants always differ.**

**…**

**If you run the hyperopt scheme for the same network/task pairing multiple times, will you arrive at the same synaptic time constants? Is the performance in anyway monotonic for the decay time with a fixed rise time?**

We have included an appendix with two results. First, to demonstrate the convergence of hyperopt with respect to synaptic time constants, we plot the distribution of rise times and fall times discovered by repeatedly running hyperopt for a single network and all four neuron models. Second, to show differences in time constants across networks and neuron models, we report a table with the mean rise times and fall times for each neuron model on each task. We direct the reader to these appendices in Sec. 4.1.

**One of the core problems I have with the procedure to optimize the time constants. Many networks are engaged in more than a single task, and don’t optimize the time constants at all, or at least through no known mechanism. In fact, there’s typically a narrow range for specific synapse types. I’m not sure of the biological plausibility of this approach.**

We have added additional discussion in Sec. 5.1 (biological plausibility) to address this concern. To summarize, the optimization of synaptic time constants was not intended to mimic a biological mechanism, but simply to find time constants that realized particular network-level dynamics given complex neuron-level dynamics. A similar technique (optimizing synaptic dynamics for network-level functionality) has been applied using similar methods (EBN) [1]; these authors treat the question of biological realism in a similar manner, but also note that “recent advances in learning temporal connections between neurons (Kennedy et al., 2014), together with learning algorithms for the present spike-based computation framework (Bourdoukan et al., 2012), provide a basis to potentially derive a learning rule for the [synaptic] filters“. There may be some optimization process in the brain that effectively selects which neurotransmitters/receptors are used in a given network, such as evolutionary selection or pruning during development. osNEF optimization via hyperopt certainly would *not* reproduce the mechanisms of such an optimization, but it may reproduce the results, with respect to the balance of time constants observed in the final network. We also note that the time constants discovered by hyperopt often fall within biologically plausible ranges (see the new table and discussion in the appendix).

[1] Schwemmer, Michael A., et al. "Constructing precisely computing networks with biophysical spiking neurons." *Journal of Neuroscience* 35.28 (2015): 10112-10134.

**Figure 3 was not labeled in the attached PDF! I’m not sure if this was down to the authors, or the submission system. The figures are also out of order at the end. I think what's happened is the figures were uploaded possibly out of order or were not explicitly labeled in the submission system with the right figure index.**

We apologize for the mixup, there were some problems with the submission system and labeling/ordering of figures.

**Equation 12, while is an oscillator, is of a very special type. It is non-hyperbolic, meaning all amplitudes for the oscillator are stable. So if simulated long enough, the amplitude should decay, or explode. I’m a bit surprised there’s stability over 100s, I would show the amplitude of the cycles at the end of the 100s.**

We have extended Fig. 9 to show stable amplitude up to 100s in all four neuron models. We also note, through an additional paragraph in the section 5.2, how the dynamical systems implemented in our neural network are only *approximated* by the ODEs given in the target equations. The exact dynamics that emerge from a network will depend on the training signals used, on the tuning curve distribution and saturation of firing rates, and other factors. We discuss situations in which these constraints (including the noise inherent in spiking representations) are helpful versus harmful for osNEF training.

**I’m not sure what the big picture here is in regards to the biophysical detail. It seems like it’s largely pointless in terms of any particular neuron being better or worse at specific tasks, as no one model really outperforms any other.**

The purpose of this paper was not to find a neuron model that outperforms other neuron models. Our primary goal was to develop a method that trains neural networks, populated with *arbitrary* neuron models, to implement specific dynamics or perform particular cognitive operations. We mention in the introduction and the discussion that such a method allows researchers to investigate questions that relate low-level biological details to high-level cognitive capacity, for instance by manipulating calcium channels and observing changes in performance on a cognitive task. To clarify this motivation, we have added additional language in the introduction and discussion, which hopefully explains the larger goals of the paper with respect to biophysical detail.

**Reservoir methods like FORCE/echo state training can also use biophysically detailed models, since the learning algorithms are agnostic towards the node dynamics used. Further, there is work performed by the Deneve group that also extends the EBN approach to Hodgkin-huxley type models with linear dynamics. So, to what extent is the training of biophysically detailed neuron models even novel anymore? This seems to be possible now with a variety of techniques.**

The reviewer is correct that recent papers using FORCE training [1] and EBN [2] have implemented linear (and sometimes nonlinear) dynamics in neural networks populated with biologically detailed neurons. We have expanded our discussion in Sec. 5.4 (Comparison to other methods) to discuss similarities and differences to these approaches, focusing in particular on what makes our approach novel. We summarize this discussion here.

Broadly speaking, there are important theoretical differences between the underlying frameworks, FORCE, EBN, and NEF. Even if we assume that all three methods solve similar problems with similar performance, we believe there is significant value in developing and presenting an NEF-based method that is comparable to FORCE- and EBN-based methods. The NEF modeling community is quite large, so validating a method for training biologically-detailed functional models within this framework has value, independent of similar successes with FORCE and EBN. Furthermore, given the complexity of training biologically-detailed networks, we believe that the research community will benefit from the existence of multiple methods that tackle this problem in different ways. We hope that future work will compare these methods, identify their relative strengths and weaknesses, and develop new methods that build upon their successes.

Second, there *are* significant differences between these three methods with respect to biological plausibility and computational capacity. In the FORCE paper, the authors simulate spiking LIF, Theta, and Izhikevich neurons, showing that they can produce a number of dynamical systems, including various oscillators, chaotic systems, songbird singing, and episodic memory. These methods use a variety of current-based synapse models (exponential, double exponential, and alpha) and respect Dale’s principle. In the EBN paper, the authors simulate point neurons that include Hodgkin-Huxley-type ionic currents, showing that they can produce several types of one-dimensional integrators that statistically reproduce empirical patterns of neural activity. Their methods also use double-exponential synapses, but do not respect Dale’s principle. In our paper, we simulate three point neurons and one pyramidal cell reconstruction with four compartments and six ionic currents. We show that our trained networks reproduce five dynamical systems (feedforward and recurrent networks for linear systems) and perform a cognitive task at performance levels comparable to simple animals. Our methods use both current-based double-exponential synapses and conductance-based, voltage-gated NMDA synapses; we do not respect Dale’s principle. We feel confident in claiming that we have (a) simulated neurons with significantly more biological realism than the FORCE and EBN papers, (b) demonstrated that our trained networks perform a wider variety of computations/tasks than the EBN paper and a comparable number to the FORCE paper.

One might argue that the greater biological realism and dynamical versatility that we achieve are incremental additions of complexity, and that the FORCE and EBN methods could also accommodate this added complexity. However, it is not immediately clear that FORCE and EBN can easily handle more biological complexity without loss of performance or the development of additional methodological tools. It is true that (a) both FORCE and EBN are agnostic about neuron model complexity, and (b) the above papers have convincingly demonstrated these methods working with moderately-complex neurons. Could these methods also accommodate the greater biological realism and dynamic versatility that we investigate in this paper? Maybe, and maybe not; supporting this claim would require running numerous experiments and comparing the results to osNEF-trained networks. We are skeptical that FORCE and EBN methods (as they currently exist) could recreate our results, for a few reasons.

- Standard NEF networks can accommodate several neuron models (ReLUs, LIFs, Izhikevich neurons, etc) without catastrophic loss of performance. Given these examples, and the fact that the NEF is agnostic about the neural processes that generate spikes within neurons, one could claim that the standard NEF supports networks containing biologically detailed neurons. However, our preliminary experiments revealed this claim to be false: if one uses standard NEF methods to train networks containing highly-adaptive or (temporally) nonlinear neurons, the resulting networks will perform very poorly. This failure was, indeed, a primary motivation for the current work; it is possible that, when faced with similar challenges, FORCE and EBN might fail in similar ways.
- FORCE training has not yet been used to train networks populated with neurons that include *temporal* nonlinearities, such as those introduced by coupled ion channel dynamics or voltage-gated synapses. We are curious to what degree this particular form of added complexity could be accommodated by the FORCE learning rules.
- EBN training with HH-type neurons required a significant theoretical extension to the original theory, notably the “inclusion of synapses with appropriate kinetics”. While both EBN and osNEF utilize synaptic dynamics to account for the nonlinear dynamics of complex neurons, these two methods rely on significantly different optimization processes for discovering those synaptic dynamics. The EBN approach uses a form of system identification that (a) drives the neuron model with a specific random process, (b) analyzes the resulting voltage traces when the neuron spikes, (c) calculates the average action potential waveform, (d) takes its temporal derivative, and (e) convolves it with an exponential function. While this method is applicable to any neuron model that generates voltage traces, we anticipate two challenges in applying it to the diversity of neuron models that we investigated. First, can this method be applied to neuron models with discontinuous voltage dynamics? Many intermediate-complexity neuron models, such as the commonly-used LIF and Izhikevich neurons, rely on instantaneous voltage reset mechanisms that may be problematic for voltage-based system identification. Second, can this method be applied to multi-compartment neuron models? Voltage can be measured and analyzed in the somatic compartment, but can this compensating synaptic kernel be used to set parameters (e.g., synaptic weights) in distal compartments? In contrast to the EBN approach, the osNEF approach relies on a black-box optimization that (a) simulates the network with task-relevant inputs, (b) collects spikes and state space targets over time, and (c) searches for synaptic time constants that, in conjunction with decoders obtained via a least-squares optimization, effectively map filtered spikes onto those targets. Because osNEF measurement and optimization occurs in spike space rather than voltage space, osNEF can be applied *without modification* to any neuron model that generates spikes.

We reiterate that FORCE and EBN are powerful and versatile tools for training neural networks, and it is entirely possible that they can accommodate the ranges of biological and dynamical complexity that we investigate in this paper. Nonetheless, we have *shown* that osNEF is capable of successfully training such systems, making this paper a novel contribution in terms of the *degree* of biological realism and functional capacity.

[1] Nicola, Wilten, and Claudia Clopath. "Supervised learning in spiking neural networks with FORCE training." *Nature communications* 8.1 (2017): 1-15

[2] Schwemmer, Michael A., et al. "Constructing precisely computing networks with biophysical spiking neurons." *Journal of Neuroscience* 35.28 (2015): 10112-10134.

**As far as I can tell, there’s no noise included in the networks here at all. Would any of the network dynamics still be trainable if the neurons each received a white noise process?**

We have added an appendix which includes an experiment that investigates this question. We took our simplest feedforward network and introduced a population of poisson spiking neurons, “poisson”, that are driven by a 10Hz band-limited white noise signal. We connected “poisson” to ”pop1” with a random weight matrix w_poisson_pop that was obtained by copying w_pre_pop and randomly shuffling the entries. By setting the maximum firing rates of “poisson'” to mirror the maximum firing rates of “pre'”, and setting w_poisson_pop to be a shuffled version of the osNEF-trained w_pre_pop, we ensured that the weighted spikes delivered to “pop1” by “poisson” would drive our detailed neurons with (approximately) the same force as a standard state space input. We then scaled w_poisson_pop by a factor gamma between zero and three: gamma=0 causes noise having no effect, gamma=1 causes noise to have (approximately) the same impact as the input x(t), and gamma>1 should cause noise to overwhelm the input signal. For each neuron model and for each value of gamma, we ran five tests with unique input signals, noise signals, and random shufflings of w_poisson_pop. We then plotted the change in RMSE between the decoded estimate from “pop2” and the state space target as a function of gamma. As expected, RMSE grows as the impact external noise becomes greater; this increase appears to be approximately linear in gamma, which is a good indication that our representational scheme is robust to external perturbation and degrades gracefully as the signal-to-noise ratio decreases.

In the discussion, we also point out that simulation with spiking neural networks inherently includes noise: the signals transmitted within the network (through weighted synapses) are represented imperfectly by the spiking activity of neurons, leading to an implicit noise component in each signal.

**I would agree that some cellular features (line 46) are not useful for information processing, I just would not explicitly include the examples of mitosis/energy transport the authors make. Mitosis for example is necessary in neurogenesis in the dentate gyrus. To what extent its regulated by information processing requirements remains to be investigated. I would just be careful here and remove the examples.**

We have replaced the example of “energy transport and mitosis” with “cellular respiration”.

**Is equation (3) the formal cross product between two vectors, or just the matrix product? I would not use the X symbol when mentioning two vectors. The encoders introduced as a tensor later on, so I would just make it explicit what this operation is.**

The “x” symbol in Equation 3 is the cross product; we have added a note that explicitly states this in the text. Later, we use the (dot) symbol to indicate a product between elements of the encoder tensor and elements of the decoder matrix; we also define this symbol in the text.

**delayed response task -> Delayed Response Task (DRT)**

We did not find any instances of “delayed response task” or “delayed match-to-sample” task being capitalized in our references, so we are leaving this as-is. We do add abbreviations (DRT and DMTST) after introducing these tasks and use these abbreviations throughout the paper, except in the abstract and conclusion.

Reviewer 2

Reviewer 2 did not request any changes to the manuscript. We thank them for their positive feedback!

Reviewer 3

Reviewer 3 requested a number of clarifications in the explanation of the methods and the presentation of the figures, especially the network diagrams. We have incorporated their specific suggestions and attempted to resolve confusions surrounding model details and the relationship between our work and standard NEF methods. In particular, we have made major revisions to Figure 2, our primary diagram for explaining the workflow of the osNEF training method, and its explanation in sections 3.2 and 3.3. These changes are focused around (a) thoroughly explaining what computational operations happen at each stage, (b) referencing what quantity / variable results after each step, and (c) including a table of equations that summarize the whole workflow.

**Network schematic figures (e.g. Figs 2,5,8,10,12) are very difficult to follow, and lack critical descriptions to understand them. For example, what is H in each? (I am left to assume this refers the standard engineering usage of H as an impulse response function, but this should be made explicit.) Figure 12 is particularly mysterious. Broadly, all of these figures are a bit on the basic side, and the model design principles are quite complex. More detailed figures would help to clarify the details of the design procedure. Perhaps adding some plots of the different signals at each stage (e.g. x, each neural population, etc.) would help make the model more clear.**

In addition to the above changes to the network schematic figures, we have included an additional equation (and explanation) of how H is used to filter spikes, which was previously mentioned only briefly in the text.

**In Fig. 4 top and middle, what are the different lines? Different model neuron classes or different neurons in a single network? It’s very difficult to parse those lines. If its different model neurons, there are 4 example model types (by my count) but it looks like there are more than 4 lines?**

Each line is the decoded estimate from a population of neurons containing the indicated neuron model; we have updated the figure caption to clarify this explanation. There are only 4 lines (plus the black state space target), as per the figure caption.

**multiple figure captions are fairly terse. For example, the Figure 7 caption doesn’t seem to be related to the actual content of Figure 7 in any way that was clear to me.**

We have expanded the figure captions of all our figures to better indicate what experiment is being run and what is being plotted.

**Several terms are introduced without explanation. What is hyperopt? (Of course I can google this myself, but shouldn’t have to). What is the PES learning rule (i.e. what does PES stand for? Never defined).**

These terms have now been defined and referenced in the text.

**The basics of the most simple model structure are difficult to follow (Section 3.2) and this is not helped by Figure 2 or the mathematical notation. a_pre is clearly designated as a presynaptic population, but what is a_pop? Is this population of neurons recurrently connected? In addition to an improved Figure 2 making the model architecture more clear, a more thorough treatment of the model details would make it more clear. Relatedly, the model equations are a bit scattered all over the place, because of the way the model is built up. It might be appropriate at 3.2 to describe the model in full detail (even revisiting things that have already been discussed) so all of the model components can be understood in relationship to each other.**

These suggestions are addressed by the overhaul of the network diagram, and the inclusion of a table of equations, described above.

**The authors seem to use the sections ‘Representation’, ‘Computation’, and ‘Application’ as anchors, but to me, this did not incrementally introduce the success of their approach, as I think the authors had intended. For example, the distinction between representation and computation was not clear to me, and the clarify was not aided by the figures. So either a restructuring of the results sections should happen or the existing structure should be explained more clearly.**

We have added some language at the beginning of the results section to help organize these sections. We especially note the differences between representation and computation.

**The online decoder learning rule is different than the NEF decoder learning rule (I think). Is this correct? If so, this should be more explicit, and also the decoder learning rule from the NEF should be stated explicitly so this distinction can be made more clear.**

The decoder learning rule is identical to PES, as we noted in the text above Equation 6. We have added new text that explains how PES is a standard NEF method, which we take as-is, but combine with a novel encoder learning rule to make an original contribution.

**It’s difficult to understand how the NEF is used (apart from inspiring the encoder/decoder convention) within the authors’ framework. Specifically, it seems that the NEF is used as a target for the encoders of the ‘learner’ network. How exactly this all works together (how is the target network constructed and how is it used for the learning network?) is difficult to follow from the manuscript (and also not helped by the figures). (Note that the comparison the authors provided in the Discussion between their approach and the full-FORCE approach of DePasquale et al, e.g. as a target generating and learner network, was helpful in bringing this key network design aspect into the light, but still, it would help to make this much more clear in any event, and especially much earlier in the manuscript than the Discussion, probably in Section 3.2).**

We have added text throughout Section 3 describing what is original NEF and what is osNEF; we have also summarized these differences in the introduction, to point out our novel contributions. These extend the existing discussion that details these differences in Section 5.4.

**One element that is a bit unique about the NEF framework, and that the authors’ framework seems to share, is that the models are often described as having the state value x input into the system and the computations are done as operations on this representation. To me, this is often a somewhat foreign perspective and differs from the more typical perspective of neural networks these days where a state variable is contained implicitly within a neural population that potentially receives other external inputs. To me, clarity could be improved by walking a bit more slowly through the model architecture and to specifically drawn attention to and clarify this novel aspect of NEF-like frameworks.**

Our added explanations and figure updates include clearer definition of, and discussion about, state space representations. However, the notion that neural activity is represented by a state variable **x**, and that network connections compute functions on this representation, is quite common in this field; notably, it is central to NEF, FORCE [1], and EBN [2] architectures.

[1] Nicola, Wilten, and Claudia Clopath. "Supervised learning in spiking neural networks with FORCE training." *Nature communications* 8.1 (2017): 1-15

[2] Schwemmer, Michael A., et al. "Constructing precisely computing networks with biophysical spiking neurons." *Journal of Neuroscience* 35.28 (2015): 10112-10134.

Thank you again for your time and consideration of our manuscript. We believe the changes we have incorporated have significantly strengthened the paper. We look forward to hearing from you. Please do not hesitate to contact us if you have any further questions.

Sincerely,

Pete Duggins and Chris Eliasmith
